# Patient-reported outcomes associated with different restorative techniques in pediatric dentistry: A systematic review and MTC meta-analysis

**DOI:** 10.1371/journal.pone.0208437

**Published:** 2018-12-06

**Authors:** Nathalia Miranda Ladewig, Tamara Kerber Tedesco, Thaís Gimenez, Mariana Minatel Braga, Daniela Prócida Raggio

**Affiliations:** 1 Department of Pediatric Dentistry and Orthodontics, Faculty of Dentistry, University of São Paulo, São Paulo, São Paulo, Brazil; 2 Department of Pediatric Dentistry, Faculty of Dentistry, University of Ibirapuera, São Paulo, São Paulo, Brazil; Kwangwoon University, REPUBLIC OF KOREA

## Abstract

**Background:**

Despite the increasing number of studies evaluating patient reported outcome measures (PROs), there is no clearness regarding which restorative treatment offers major benefits based on the pediatric patient perspective.

**Aim:**

To compare different restorative techniques in pediatric dentistry regarding patient-reported outcomes.

**Design:**

Literature searching was carried out on prospective studies indexed in PubMed, Scopus and OpenGrey. A Mixed Treatment Comparisons (MTC) meta-analysis was undertaken considering the results from reviewed studies. Anxiety, pain and quality of life were extracted as mean with standard deviation, percentage of pain, and mean difference of scores with standard deviation, respectively. For direct comparisons, data were combined using a random-effect model. Heterogeneity was assessed with the I^2^ statistic. For indirect comparisons, fixed and random effects were chosen through comparison of competing models based on the Deviance Information Criteria (DIC). The expected efficacy ranking based on the posterior probabilities of all treatment rankings was also calculated.

**Results:**

An initial search resulted in 4,322 articles, of which 17 were finally selected. Due to unavailability of data, only pain, anxiety and oral health related quality of life (OHRQoL) were statistically analyzed. The difference in means (95% CI) of anxiety between treatments using only hand instruments with or without chemomechanical agents were -5.35 (-6.42 to -4.20) and -5.79 (-7.77 to -3.79) respectively when compared to conventional treatment using rotary instruments and/or local anesthesia. Regarding pain, there was a trend for treatments without rotary instruments and local anesthesia to be less frequently reported as painful. No statistical difference was found intragroup nor among treatments for OHRQoL.

**Conclusions:**

Anxiety and pain are directly related with more invasive restorative treatments. On the other hand, quality of life is not improved regardless of the restorative technique used. Further well-designed prospective studies regarding PROs in children are still necessary.

## Introduction

Patient-reported outcome (PRO) is an assessment of health status reported by the patients themselves instead of being interpreted by an observer[1]. Although physical, physiological and biomechanical data may be measured through medical examination, there is some information that can only be obtained from the patient, such as symptoms, feelings and disease’s impact[2]. The utilization of PRO in health care is an emerging metric that is becoming increasingly important[3–5]. It is considered an essential component in the provision of health care and ensures patient’s voice and his engagement in medical decision-making[6]. In the field of pediatric dentistry, this takes a major role because negative dental experiences during childhood and adolescence reverberate in adulthood, presenting a long-term effect[7].

As restorative care is a fundamental part of the comprehensive oral health treatment of children[8], it has been more extensively studied regarding PROs. The traditional clinical parameters to assess restorative treatments, such as marginal integrity and wear surface of restorations, secondary caries, and pulp inflammation[9], have been complemented by patient-based outcomes as quality of life[10,11], anxiety[12–13], children and parental perception[14] and pain[14–18].

Recent studies have been focusing on patient-based outcomes measured not only by psychometric scales[19–21] but also physiological rates or under the health professional perspective[19]. However, the published data presents conflicting results and inconclusive findings, with no clearness regarding which restorative treatment offers major benefits based on the patient perspective. In this context, the aim of this systematic review is to compare different restorative techniques in pediatric dentistry regarding patient-reported outcomes.

## Material and methods

This systematic review was reported according to PRISMA guidelines[22] as detailed in Supporting Information section. A review protocol was recorded at PROSPERO database under the registration number CRD42017056285.

### Literature search

The literature search on MEDLINE (PubMed), Scopus and OpenGrey was performed until February 2018. A search strategy was developed for MEDLINE (PubMed) and then suited to the other two databases (Table 1). Three groups of words combined with the boolean term ‘OR’ were created, including key words for primary teeth, restorative treatment and patient-based outcome. The three groups were combined with the boolean term ‘AND’. Both Text Word and Mesh Terms were used. Hand searching was performed on reference lists of full-text read articles and no languages restrictions were applied.

**Table 1:** Search strategy developed for MEDLINE via PubMed.

#1	(child*) or (children) or (pediatric) or (paediatric) or (infant*) or (minor*) or (deciduous tooth) or (deciduous teeth) or (primary tooth) or (primary teeth) or (primary dentition) or (baby tooth) or (baby teeth) or (primary molar*) or (adolescent*) or (adolescent) or (deciduous tooth) or (deciduous teeth) or (deciduous dentition) or (primary tooth) or (primary teeth)
#2	(restorative treatment*) or (dental restoration*) or (dental filling*) or (atraumatic restorative treatment, dental) or (atraumatic restorative treatment) or (amalgam) or (resin composite) or (composite resin) or (composite restoration*) or (compomer) or (glass ionomer cement) or (permanent dental restoration*) or (permanent dental filling)
#3	(pain) or (discomfort) or (anxiety) or (quality of life) or (fear) or (patient based outcome) or (patient centered outcome) or (patient satisfaction) or (dental fear) or (dental phobia) or (odontophobia) or (panic) or (acceptability) or (tooth appearance) or (oral health related to quality of life)
#4	#1 AND #2 AND #3

* Truncating search terms: it finds terms that begin with the word’s root

### Selection criteria

Eligible studies in this systematic review included prospective studies assessing dental restorative treatment in the primary dentition. The lack of a comparison group and patient-reported outcomes as well as studies performed in groups with specific conditions different from normality and studies in which primary and permanent dentition data were not analyzed separately were excluded. As patient-reported outcomes we consider all assessments that are reported by the patient according to the CONSORT PRO Extension[1]. In addition, the proxy-reported outcome Oral Health Related Quality of Life (OHRQoL) was also appraised.

### Review methods

Titles and abstracts were screened independently by two reviewers (N.M.L and T.K.T.). If the study met the inclusion criteria or if there were insufficient data available, full-text articles were obtained for further assessment by the same reviewers. Cohen Kappa test was performed to ensure their inter-rater reliability before both phases using 10% of the search sample. Disagreements were discussed with an expert (D.P.R.) to reach consensus.

### Data extraction and processing

Relevant data were collected using a structured data extraction form. Author, publication year, country, study location and design were extracted to describe the studies. Sample size, age of participants, group of teeth treated and sample size according to the treatment were collected to characterize the sample. Regarding the results, operator, outcome, evaluation criteria, time of evaluation and main findings were extracted. Authors of included studies were contacted to provide additional data when needed.

The treatments compared among the studies were categorized in six groups according to the characteristics described: I- Restorative treatment using rotary instruments and local anesthesia (BUR+LA); II- Restorative treatment using rotary instruments (BUR); III- Restorative treatment using hand instruments and adhesive material (HI); IV- Restorative treatment using chemomechanical agents (CHM); V- Hall Technique (HT); VI- Ultraconservative restorative treatment (UCT).

Studies’ quality was assessed by two reviewers (N.M.L and T.K.T) independently. The Cochrane Collaboration Tool was used to appraise all studies included. Each study was evaluated as low, high or unclear risk of bias according to the randomization, allocation concealment, blinding, completeness of outcome data, selective outcome reporting, and other potential bias. Authors were contacted via e-mail for missing or unclear information. Disagreements between the reviewers were solved by consensus.

Publication bias would be assessed if more than 10 studies were identified, since power is low otherwise [23].

### Statistical analysis

The three outcomes quantitatively evaluated in the meta-analysis were considered as continuous variables and treated according to the measures available in each article. Mean with standard deviation, percentage of pain, and mean difference of scores with standard deviation were extracted to evaluate anxiety, pain and quality of life respectively. The coefficients reported in the meta-analysis were difference in means with 95% confidence interval (95%CI) for anxiety and quality of life and risk relative (RR) with 95%CI for pain.

The effects of each treatment for dental caries in primary teeth on patient-reported outcomes were analyzed using a Mixed Treatment Comparisons (MTC) meta-analysis. The MTC combines direct and indirect comparisons across a range of competing interventions by including multiple distinct pairwise data. It also allows the calculation of treatment ranking probabilities regarding their efficacy[24]. As MTC is based on Bayesian hierarchical framework, the estimates were obtained by Markov-Chain Monte Carlo simulations. All analysis was performed in the R statistical software using the GeMTC package version 0.8 and the rJAGSpackage to estimate the models.

For direct comparisons, data were combined using a random-effect model. Heterogeneity was assessed with the *I*^*2*^ statistic when more than one study compared the same treatments regarding the same outcome.

For indirect comparisons, the choice between fixed and random effects was made through the comparison of competing models based on the Deviance Information Criteria (DIC). For each model, goodness-of-fit to data was evaluated using residual deviance[25]. Vague prior distributions were used for all models. The expect ranking of efficacy for all treatments based on the posterior probabilities of all treatment rankings[26] was also calculated. Node split analysis for inconsistency was not performed because most part of the treatments did not present direct comparisons.

## Results

**Fig 1:**
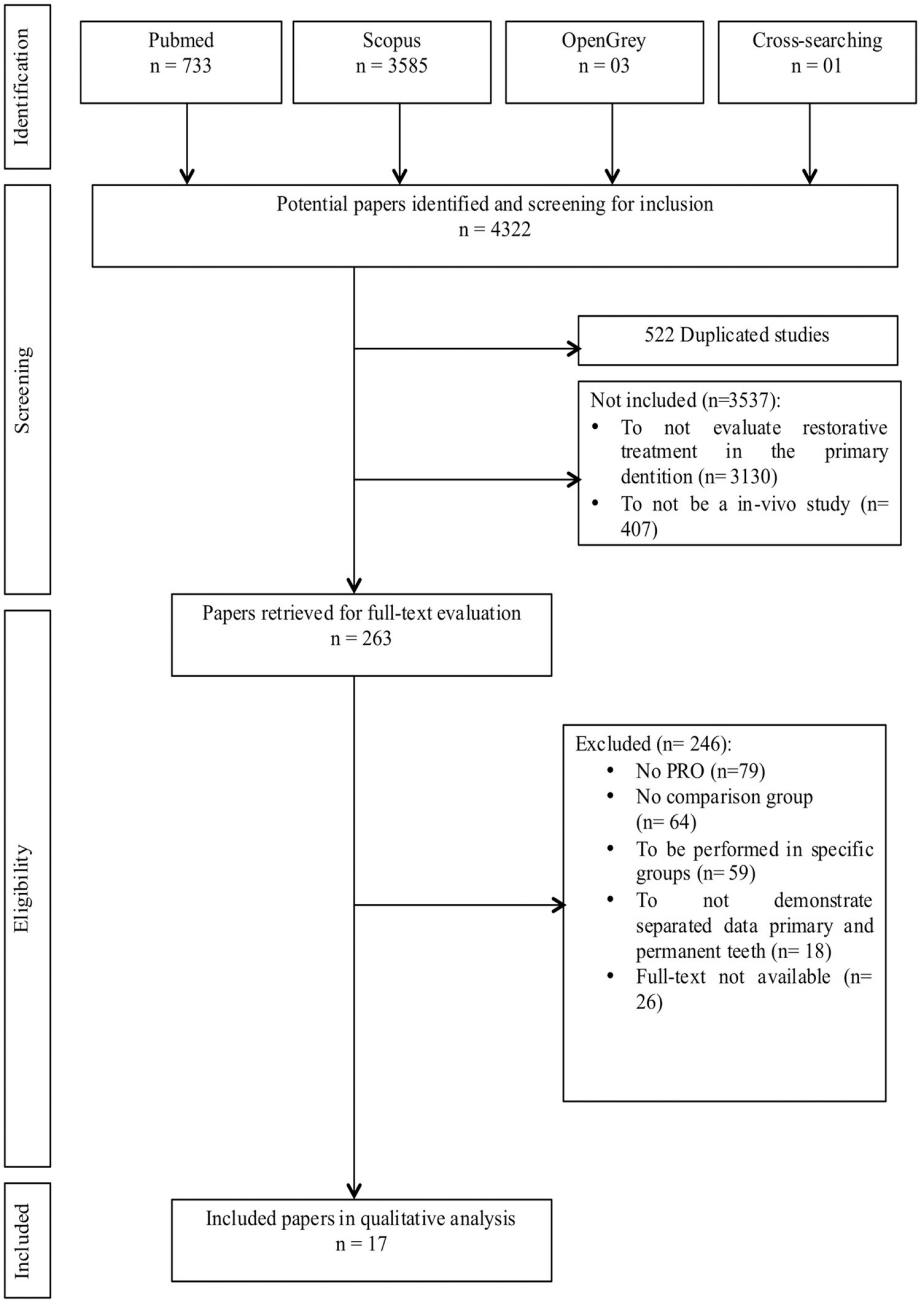
Flowchart of studies selection.

In total, 4,322 studies were identified through the search strategy of which 3,800 were non-duplicated. The inter-rater reliability was 0.79 for abstract inclusion and 1.0 for full-text exclusion. After screening titles and abstracts, 263 papers were retrieved for full-text evaluation. The main reason for excluding studies was the absence of patient-reported outcome measures (n = 79). A final number of 17 papers met the eligibility criteria (Fig 1). From those, eight different patient-reported outcomes were identified as following: pain (n = 7), discomfort (n = 2), treatment preference (n = 1), anxiety (n = 6), quality of life related to oral health (n = 2), satisfaction (n = 3), willingness to receive the treatment again (n = 1) and appearance (n = 1). Some papers reported more than one outcome (Table 2).

**Table 2:** Main characteristics of data from included studies.

Author/ Year and Country	Designesign	Location	n (patient)	Age (years)	Teeth	N in according to the treatment	Operator	Outcome	Evaluation criteria	Moment of evaluation	Findings
Louw et al. [15] 2002 (South Africa)	RCT^†^ Parallel Groups	Clinic	401	6–9	Primary teeth	ART*: 175 ART* w/ bur: 117 ART* & ART* w/ bur: 109	Dentists	Pain	Question: Did you feel pain when the tooth was being fixed?	10 days after treatment	**% Pain** ART*: 19 ART* w/ bur: 15 ART* & ART* w/ bur: 22
Bochove et al. [27] 2006 (Suriname)	RCT^†^ Parallel Groups	Clinic	300	6–7	Primary molars (Proximal cavity)	Bur–n/m ART* –n/m Bur w/ LA^§^ –n/m ART* w/ LA^§^- n/m	Final year dental student and dentist	Discomfort	Venham Picture Test	Immediately after treatment	No numerical results available
Innes et al. [28] 2007 (Scotland)	RCT^†^ Split mouth	Clinic	132	3–10	Primary molar	Bur w/ LA^§^: 132 Hall Technique: 132	General clinicians	Child Preference	Question	After completion of both treatments	**% Most preferred** Children: 72% Hall Technique
Mickenautsch et al. [12] 2007 (South Africa)	RCT^†^ Parallel Groups	Clinic	143	8.9 (Mean) 10.3 (Mean)	Primary teeth	ART*: 59 Bur w/ LA^§^- 84	Dental operators	Anxiety	Children’s Fear Survey Schedule	Immediately after treatment	**Mean Score (SE**^π^**)** A: 24.4 (1.2) B: 14.8 (1.7)
Topaloglu-Ak et al. [13] 2007 (A) (Turkey)	RCT^†^ Parallel Groups	Clinic	518	6–7	Primary molar (Proximal cavity)	Bur: 64 ART*: 96	Dentists	Anxiety	Venham Picture Test	Immediately after treatment	**Mean (SD**ˆ**)** Bur: 1.0 (1.6) ART*: 1.0 (1.7)
Topaloglu-Ak et al. [13] 2007 (B) (Turkey)	RCT^†^ Parallel Groups	Clinic	518	6–7	Primary molar (Proximal cavity)	ART*: 158 ART* w/ Cariosolv: 150	Dentists	Anxiety	Venham Picture Test	Immediately after treatment	**Mean (SD**ˆ**)** ART*: 1.7 (1.9) ART* w/ Cariosolv: 1.3 (1.7)
Abreu et al. [16] 2009 (Brazil)	RCT^†^ Parallel Groups	Clinic	40	4–7	Primary molars	ART*: 20 Bur w/ LA^§^: 20	Not mentioned	Pain	Wong-Baker Facial Scale	Immediately after treatment	**% Pain** ART*: 7 Bur w/ LA^§^: 25
Aguilar et al. [17] 2012 (Peru)	RCT^†^ Split mouth	Clinic	30	5	Primary molars (Occlusal cavity)	ART* w/ Papacarie: 30 Bur w/ LA^§^: 30	General clinician	Pain	Wong-Baker Facial Scale (Yes/No)	Immediately after treatment	**% Pain** ART* w/ Papacarie: 3.33 Bur w/ LA^§^: 53.33
Luz et al. [18] 2012 (Brazil)	RCT^†^ Parallel Groups	Clinic	30	4–7	Primary molars (Proximal cavity)	ART*: 15 Bur w/ LA^§^: 15	Not mentioned	Satisfaction Pain Willing to receive the treatment again	Facial Image Scale (FIS) Yes/No Question: Would you be prepared to receive the same type of treatment again?	Immediately after treatment	ART*: 50% Satisfied, 42.9% Pain, 37.5% Willing Bur w/ LA^§^ 2% Satisfied, 62.5% Pain, 71.4% Willing
Leal et al. [10] 2013 (Brazil)	RCT^†^ Parallel Groups	Clinic	277	6–7	Primary molars	Bur: 55 ART*: 47 UCT^Δ^: 58	Paedodontists	Quality of life related to oral health	B-ECOHIS	Baseline and follow-up (1 year)	**Difference in mean B-ECOHIS scores (SE**^π^**)** Bur: -0.04 (0.11) ART*: 0.01 (0.11) UCT^Δ^: -0.10 (0.11)
Mustafa et al. [29] 2013 (United Kingdom)	Prospective PROM study	Clinic	125	5–17	Primary teeth	Preformed Crown w/ LA^§^: 10 Bur w/ LA: 16	Not mentioned	Pain	FPS-R (5-7yrs) VAS (7-18yrs)	2, 4, 6 Hours after treatment	**% Pain (Highest score)** Crown: 40 Bur w/ LA^§^: 38
Santamaria et al. [14] 2014 (Germany)	RCT^†^ Parallel Groups	Clinic	169	3–8	Primary molars (Proximal cavity)	Bur w/ LA^§^: 65 Hall Technique: 52 NRCT^+^ w/ bur: 52	Paedodontists Post-graduate paediatric students	Pain	Visual analogue pain scale	Immediately after treatment	**% Pain** Bur w/ LA^§^: 42 Hall Technique: 81 NRCT: 88
Arrow et al. [11] 2016 (Australia)	RCT^†^ Parallel Groups	Clinic	254	3.8 (Mean)	Primary teeth	ART* w/ Bur: 127 Bur w/ LA^§^: 127	Dental therapists	Quality of life related to oral health	ECOHIS	Baseline and follow up (6–12 months)	**Mean (SD**ˆ**)** ART* w/ Bur: 2.00 (0.92) Bur w/ LA^§^: 1.28 (0.63)
Arrow et al. [30] 2017 (Australia)	RCT^†^ Parallel Groups	Clinic	254	> 6	Primary teeth	ART*: 127 Bur w/LA: 127	Dental therapists (ART) Dentists (Bur)	Anxiety	Facial Image Scale (FIS)	Baseline and follow up (12 months)	**% Anxiety improvement** ART*: 26 Bur: 26
Maciel et al. [31] 2016 (Brazil)	Mixed-method study	Clinic or Schools	1045	4–8	Primary molars	Hall technique: 234 ART*: 408 Amalgam: 198 Composite resin: 205	Dentists	Satisfaction (Quantitative data)	Face scale with 5 possible answers (Quantitative data) Content analysis (Qualitative data)	Immediately after treatment	**Satisfaction (%)**: Hall technique: 95.3 ART*: 94.9 Amalgam: 97.5 Composite resin: 99
Lakshmi et al. [32] 2018 (India)	RCT^†^	School	30	5–8	Primary molar	ART*: 15 Hall Technique: 15	Not mentioned	Satisfaction Discomfort Appearance	Yes/No Wong-Baker Facial Scale Positive/Negative	After treatment	**%Satisfied**: ART* 86.67 *versus* HT^+^ 66.67 **Mean Discomfort (SD**ˆ**)**: ART* 1.87 (0.92) *versus* HT^+^ 0.53 (0.92) **%Positive appearance**: ART* 100 *versus* HT^+^ 20
Barreto et al. [33] 2017 (Brazil)	Analytical cross-sectional study	School	94	6–8	Deciduous molar	ART*: 46 SDF: 48	Not mentioned	Anxiety	Facial Image Scale (FIS)	Before, during and after treatment	**No anxiety (% Worse scenario)** ART*: 41.3 SDF: 39.8
Tavares et al. [34] 2018 (Brazil)	RCT^†^ Split mouth	Clinic	79	5–8	Primary molars	ART*: 79 Bur: 79	Pediatric dentist	Anxiety Pain	Facial Image Scale (FIS) Wong-Baker Facial Scale	Before treatment At the end of the restoration	**Median Anxiety (IQR”)**: ART* 2.0 (1.25) *versus* Bur 2.0 (1.5) **Median Pain (IQR”)**: ART* 0 (2.0) *versus* Bur 2.0 (2.0)

^†^RCT: Randomized Clinical Trial;

*ART: Atraumatic Restorative Treatment;

^§^LA: Local Anesthesia;

^Δ^UCT: Ultraconservative Treatment;

^+^NRCT: Non-Restorative Caries Treatment;

^+^HT: Hall Technique;

^π^SE: Standard Error;

ˆSD: Standard Deviation;

“IRQ: Interquartile range; n/m: not mentioned.

Cells in grey: studies that were not included in the quantitative analysis due to an impossibility to merge and compare the available data.

All studies except three, which was a prospective PRO study, a mixed-method study and an analytical cross-sectional study, were randomized clinical trials (RCT). Treatments were only performed in posterior teeth.

### Risk of bias assessment

**Fig 2:**
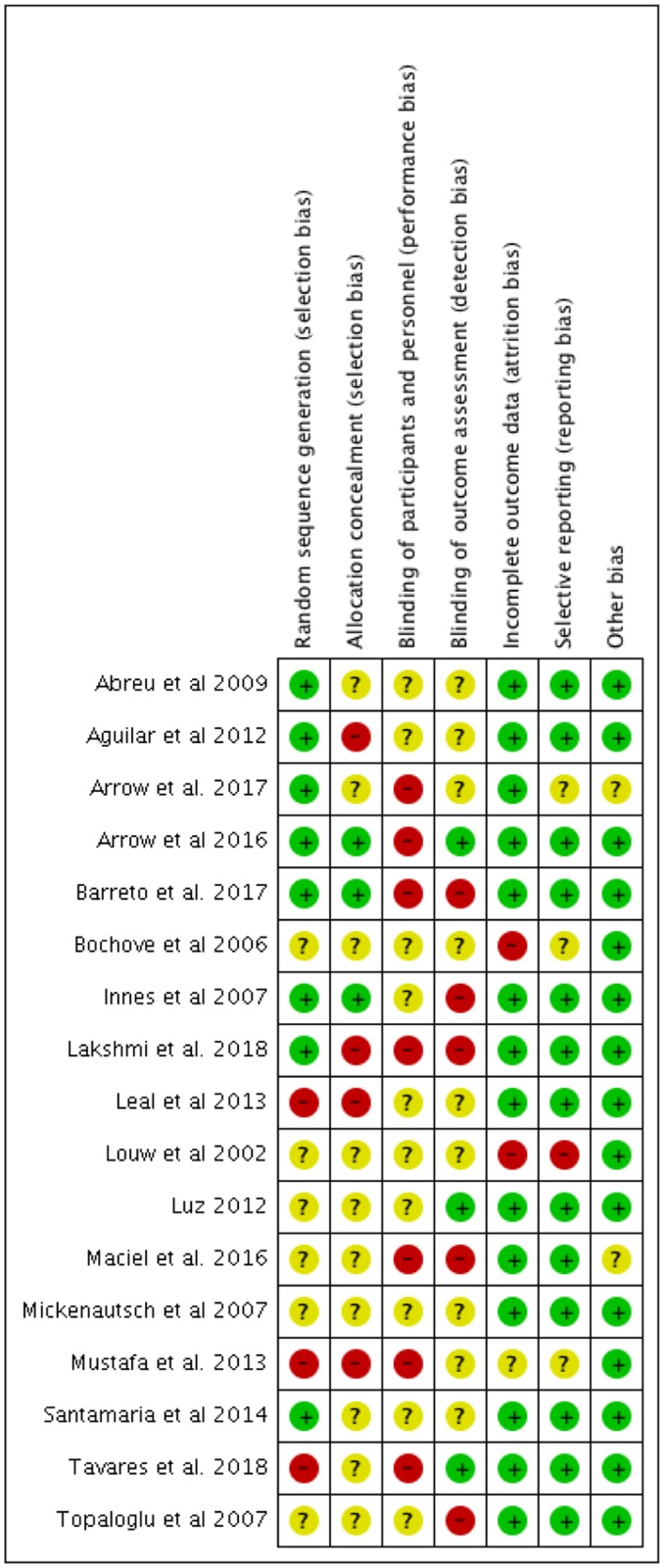
Risk of bias assessment using the Cochrane Collaboration Tool.

The assessment of the risk of bias for the included studies is displayed in Fig 2. None of the categories was classified as low risk for all the studies. Most of them did not report enough data regarding allocation concealment and blinding of participants, operators and evaluators. Reporting, attrition and selecting bias were the most frequent available low-risk bias information.

### Mixed treatment comparisons

Due to the unavailability of data regarding the same outcome, only pain, anxiety and oral health related quality of life were statistically analyzed.

#### Anxiety

**Fig 3:**
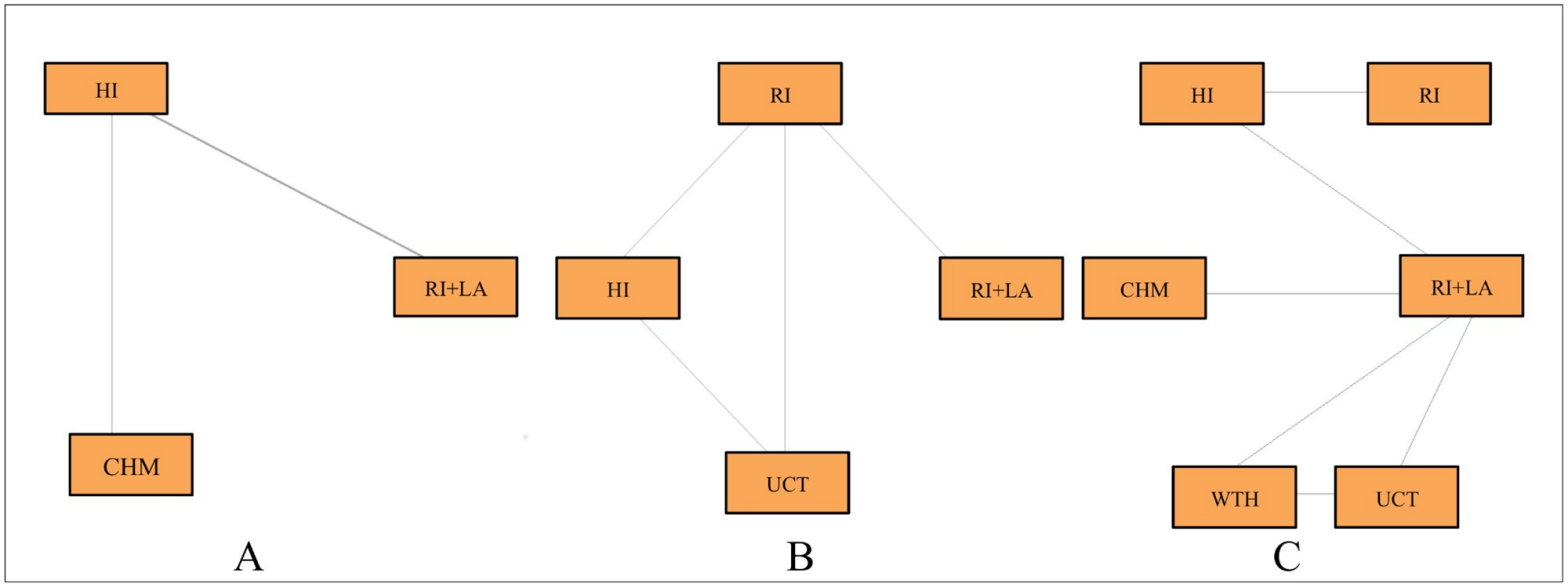
Network of the comparisons of restorative treatments in primary teeth. The width of lines connecting each pair of treatment is proportional to the number of studies regarding anxiety (A), quality of life (B) and pain (C).

From the six studies assessing anxiety, three of them presented comparable data. The Venham Picture Test was used as the evaluation tool by two studies while the Child’s Fear Survey Schedule, by the remaining one. The treatments compared were restorative treatment using rotary instruments and local anesthesia (BUR+LA), restorative treatment using rotary instruments (BUR), restorative treatment using hand instruments and adhesive materials (HI), and restorative treatment using chemomechanical agents (CHM). The mean and standard deviations were used to perform the MTC meta-analysis. Treatments (BUR+LA) and (BUR) were merged and analyzed as the same group (BUR+LA). Direct comparison was possible between treatments (BUR+LA) and (HI) as well as (HI) and (CHM) as illustrated in Fig 3A. Treatments (BUR+LA) and (CHM) were compared indirectly using a fixed effects model.

The results of the MTC meta-analysis are summarized in Table 3. Regarding anxiety, the indirect comparison identified a significant difference between treatments using rotary instruments with or without local anesthesia (BUR+LA) and chemomechanical agents (CHM). It also indicated a better performance of treatments using hand instruments and adhesive materials (HI) compared to (BUR+LA). The difference between (HI) and (CHM) were not confirmed in this model.

**Table 3:** Mixed treatment comparison (MTC) model comparing anxiety among treatments.

Anxiety	Direct comparison *	Indirect comparison **
	Difference in means (95% CI)	
HI vs. BUR+LA	-1.21 (-3.66 to 1.1)	-5.35 (-6.42 to -4.20)
CHM vs. BUR+LA	--	-5.79 (-7.77 to -3.79)
CHM vs. HI	-0.40 (-0.80 to 0.002)	-0.45 (-2.15 to 1.30)

* Random effects model,—Inverse variance method; DerSimonian-Laird estimator for tau^2; I^2 = 98.7% (97.3%–99.4%).

** Fixed effects model, Model fit: residual deviance; DIC = 19.44. Each chain used 80,000 iterations with a burn-in of 10,000 and a tinning interval of 20.

Negative values represent a decrease in the anxiety levels. Highlighted cells represent contributions from indirect comparisons.

Table 4 presents the ranking of treatments according to their probability of being the best choice. The order of the probability of less-anxiety provoking treatments was restorative treatment using chemomechanical agents (CHM), restorative treatment using hand instruments and adhesive material (HI) and rotary instruments with or without local anesthesia (BUR+LA). The probability of treatment (BUR+LA) be the worse treatment regarding anxiety was approximately 97%.

**Table 4:** Ranking of efficacy among treatments regarding anxiety.

Treatments	Position 1	Position 2	Position 3
BUR+LA	0.00025	0.02616	**0.97358**
HI	0.49933	**0.57066**	0.00000
CHM	**0.57041**	0.40316	0.02641

Bold values are the highest values in the selected columns.

#### Oral health related to quality of life

From the two studies evaluating the Oral Health Related to Quality of Life (OHRQoL), both of them used the Early Childhood Oral Health Impact Scale (ECOHIS) questionnaire. The treatments compared were restorative treatment using rotary instruments and local anesthesia (BUR+LA), restorative treatment using rotary instruments (BUR), restorative treatment using hand instruments and adhesive material (HI), and ultraconservative restorative treatment (UCT) as demonstrated in Fig 3B. The mean difference of OHRQoL scores after and before treatment and the standard deviation were used to perform the MTC meta-analysis. Direct comparison was possible between treatments (BUR+LA) and (BUR), (BUR) and (HI), (BUR) and (UCT), and (HI) and (UCT). Treatments (BUR+LA) and (HI) as well as (BUR+LA) and (UCT) were indirectly compared using a fixed effects model.

Regarding the improvement in the OHRQoL, no statistical difference was observed intragroup nor among treatments. The significant difference reported in the studies whose values did not overcome 1 was lost after performing the MTC meta-analysis.

In relation to the ranking probability of OHRQoL improvement (Table 5), treatment (UCT) was ranked in the first position even though no restorative material is used to fill the cavities in this technique. It would be followed by (BUR), (HI) and (BUR+LA). The last one presented approximately 70% of chance to be the least effective.

**Table 5:** Ranking of efficacy among materials regarding OHRQoL.

Treatments	Position 1	Position 2	Position 3	Position 4
BUR+LA	0.2382	0.0268	0.0341	**0.7009**
BUR	0.2065	0.3723	0.3364	0.848
HI	0.1153	0.2648	0.4522	0.1677
UCT	**0.4400**	0.3361	0.1773	0.0466

Bold values are the highest values in the selected columns.

#### Pain

From the seven studies assessing pain, six of them presented enough data for a quantitative analysis. Regarding the evaluation criteria, two studies used a yes/no question, two of them applied the Wong-Baker facial scale and two studies used the Visual Analogue Pain Scale. Both scales are 5-point measurements whose results were dichotomized as presence and absence of pain. The treatments compared were restorative treatment using rotary instruments and local anesthesia (BUR+LA), restorative treatment using rotary instruments (BUR), restorative treatment using hand instruments and adhesive material (HI), restorative treatment using chemomechanical agents (CHM), hall technique (HT) and ultraconservative restorative treatment (UCT). The relative risk (RR) was calculated using the percentage of pain reported for each treatment. The direct comparisons are illustrated in Fig 3C. The remaining comparisons were performed indirectly using a fixed effects model. There was only one study per pair of comparison. No statistical difference was found between treatments.

In relation to the ranking probability regarding pain, treatment (CHM) would be ranked as the low painful treatment while treatment (HT) would be the most painful (Table 6). However, the probabilities are low, 58% and 39% respectively.

**Table 6:** Ranking of efficacy among treatments regarding pain.

Treatments	Position 1*	Position 2	Position 3	Position 4	Position 5	Position 6*
BUR+LA	0.0010	0.0257	0.0632	0.2955	0.3365	0.2779
BUR	0.2161	0.3185	0.2534	0.0721	0.0571	0.0825
HI	0.1517	0.3983	0.2920	0.0709	0.0587	0.0281
CHM	**0.5869**	0.1203	0.2214	0.0359	0.0225	0.0129
HT	0.0171	0.0549	0.0790	0.1848	0.2702	**0.3937**
UCT	0.0270	0.0821	0.0907	0.3405	0.2548	0.2046

Bold values are the highest values in the selected columns.

**Fig 4:**
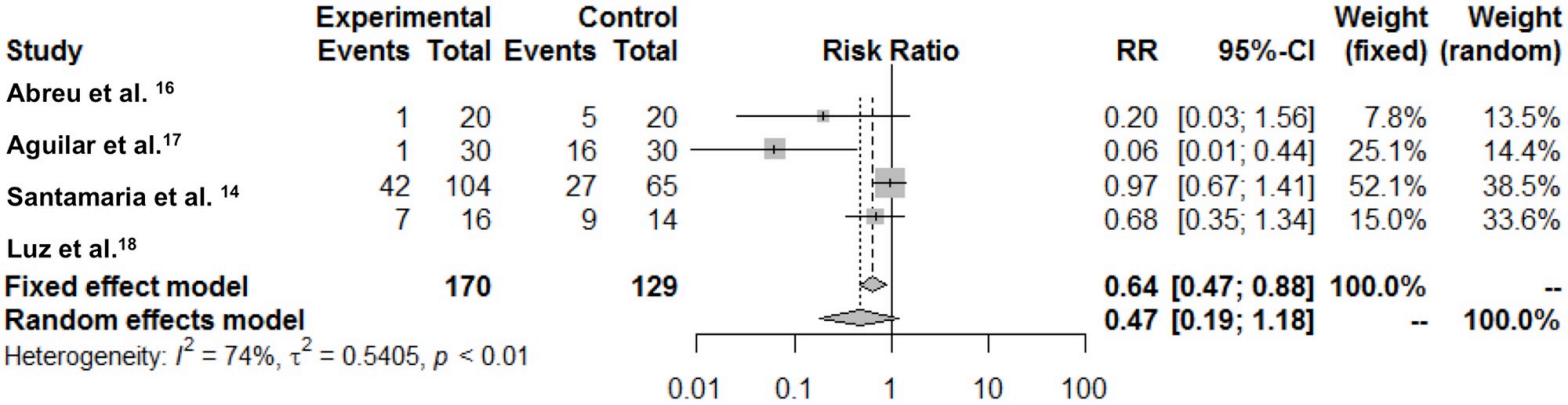
Forest plot comparing treatments with versus without rotary instruments and local anesthesia regarding self-reported pain.

A direct meta-analysis was additionally performed after dichotomizing the groups in treatments using rotary instruments and local anesthesia versus treatment without the use of rotary instruments or local anesthesia (Fig 4). From the 7 studies included in the quantitative analysis, only 4 compared dichotomized groups. High heterogeneity was found (I = 74%, 95%CI 27.6%; 90.7%), thus the random model was considered the best choice. Although no association was found using the random model, it seems to be a trend for treatments without rotary instruments and local anesthesia to be less reported as painful.

## Discussion

Despite the historical limited development and utilization of patient-reported outcome (PROs) in clinical dentistry, some important steps have been taken to report data regarding PROs[35]. This evolution seems logic considering that unpleasant dental experiences have a large range of consequences since patient apprehension[36] until dental treatment avoidance[37]. Regarding the young population, children aging 3 years and older are able to effectively communicate emotional and physical experiences despite the clear differences in their developmental skills[38] which enable them to participate in the decision-making process. In this context, it is clear to understand why the sample of all included studies were composed by children from 3 years old and up even though no age-related exclusion criteria were applied. Considering the availability of evidence-based restorative treatment options[39] and the shorter lifetime of the primary dentition, choosing a patient-friendly treatment may be the key for a successful dental management of this population.

Studies have demonstrated that there is a weak to moderate agreement between professional and children regarding some patient-based outcomes, such as pain and anxiety[40–41] and a moderate concordance between parents and children[42]. Hence, clinical observation or parents-reported measures are considered unreliable methods[43–44]. On the other hand, valid and trustworthy information can be obtained from both parents and children when measuring OHRQoL[45]. This justifies the exclusive use of patient-reported studies, excepting those regarding OHRQoL, in this systematic review.

Unfortunately, the increasing number of studies did not imply in strong and conclusive evidence. The risk of bias assessment demonstrated the lack of rigor in the publications reporting patient outcomes measures. In most of the clinical studies, it lacks information about allocation concealment and blinding of outcome assessment, parameters which could greatly influence a self-reported outcome.

The great variability of treatments and measurements regarding the same outcome limited to merger the data. Therefore, it was not possible to fully perform the MTC, since there was no direct and indirect evidence for pairwise comparison of neither outcome. However, the MTC still contributed significantly with the results, as not only increased the possibilities of comparisons among treatments for all three outcomes, but it also detected differences that had not been observed in the direct analysis of anxiety. Children reported significant higher levels of anxiety when treatments using local anesthesia and rotary instruments were performed. This result does not corroborate with a recent systematic review which demonstrated that there is no difference between ART and conventional treatment regarding this outcome[19]. However, this study only performed direct comparisons which is not enough to detect this difference with the current scientific data available.

The higher levels of anxiety related to conventional restorative procedures can be explained by the use of high-speed handpiece with or without needle/anesthesia (BUR+LAR) which are triggering factors often related to adverse emotional reactions in children in the dental office[46]. In this context, less invasive treatments such as ART (HI) can be indicated as first choice treatment because it does not require the use of these devices[47].

Regarding pain, when dichotomizing treatments between those that use or do not use rotary instruments and local anesthesia, a trend in favor of procedures that do not use these devices was found. However, it was observed through the Forest Plot that the studies showing a protective effect have smaller sample sizes. Due to the clinical heterogeneity of pain, it is possible that the protective effect is attenuated in larger studies.

On the other hand, some results regarding both pain and quality of life that had presented statistical difference in the direct analysis lost the significance in the indirect comparisons. This may represent only a statistical effect, since indirect analysis enlarge the confidence interval as a result of the mathematical modeling[24]. However, it may also be related to the impossibility of generalizing the studies’ findings. The few data available are insufficient to come to a conclusion about this divergence.

There is an evident need for high-quality prospective PRO studies in restorative pediatric dentistry. Randomized clinical trials using PRO as primary outcomes after performing an appropriate and powerful sample size calculation could bring important contributions to the current scientific literature. Yet, following a protocol for reporting PRO[1] may improve the quality of data produced. Based on the current evidence, we can conclude that anxiety and pain are directly related with more invasive restorative treatments. On the other hand, quality of life is not improved regardless the type of restorative treatment. Further well-designed prospective studies regarding PROs in children are still necessary.

## Supporting information

S1 Table PRISMA checklist. (DOC)
